# Hug sign in intraprocedural cone-beam-CT to predict short-term response to combined treatment of hepatocellular carcinoma

**DOI:** 10.1007/s11547-024-01805-y

**Published:** 2024-03-21

**Authors:** Roberto Iezzi, Alessandro Posa, Iacopo Valente, Andrea Contegiacomo, Maria Assunta Zocco, Maurizio Pompili, Brigida Eleonora Annicchiarico, Francesca Romana Ponziani, Michele Basso, Shraga Nahum Goldberg, Felice Giuliante, Antonio Gasbarrini, Evis Sala, HepatoCatt Study Group, Enza Genco, Gabriella Brizi, Francesco Cellini, Laura Riccardi, Nicoletta De Matthaeis, Marco Biolato, Luca Miele, Lucia Cerrito, Fabrizio Pizzolante, Antonio Grieco, Gian Ludovico Rapaccini, Giuseppe Marrone, Matteo Garcovich, Alfonso Wolfango Avolio, Francesco Ardito, Gabriele Spoletini, Salvatore Agnes, Maria Vellone

**Affiliations:** 1 Department of Diagnostic Imaging, Oncologic Radiotherapy, and Hematology, Fondazione Policlinico Universitario A. Gemelli IRCCS, L.Go A. Gemelli 8, 00168 Rome, Italy; 2 Università Cattolica del Sacro Cuore, Rome, Italy; 3 Department of Medical and Surgical Sciences, Fondazione Policlinico Universitario A. Gemelli IRCCS, Rome, Italy; 4 Division of Image-Guided Therapy, Department of Radiology, Hadassah Hebrew University Medical Center, Jerusalem, Israel; 5 U.O.C. Radiologia d’Urgenza e Interventistica, Dipartimento di Diagnostica per Immagini, Radioterapia Oncologica ed Ematologia, Fondazione Policlinico Universitario A. Gemelli IRCCS, L.go A. Gemelli 8, 00168 Rome, Italy

**Keywords:** Hepatocellular carcinoma, Combined treatment, Ablation, Chemoembolization, Response prediction

## Abstract

**Objectives:**

Combined treatment of ablation and chemoembolization for hepatocellular carcinoma represents a promising therapy to increase treatment efficacy and improve patient survival. The “hug sign” is a recently introduced radiological sign consisting in deposition of beads/contrast agent during transarterial chemoembolization in the hyperemic area surrounding the post-ablation volume, seen during intraprocedural unenhanced cone-beam CT, that may indicate intraprocedural success. Aim of our retrospective study was to analyze the usefulness of the “hug sign” at the intraprocedural unenhanced cone-beam CT as an early predictor of response to combined treatment, based on the hug sign angle.

**Materials and methods:**

Between January 2017 and September 2021 all patients with hepatocellular carcinoma which underwent a combined treatment of thermal ablation followed by chemoembolization were enrolled. All treated patients underwent immediate post-procedural unenhanced cone-beam CT to evaluate the deposition of contrast agent, lipiodol or radiopaque beads and to assess the percentage of coverage of the ablated area with the contrast agent (hug sign angle). Patients with missing pre-procedural, intra-procedural and/or post-procedural data/imaging, or with poor-quality post-procedural cone-beam CT images were excluded.

**Results:**

128 patients (mean age, 69.3 years ± 1.1 [standard deviation]; 87 men) were evaluated. Our study evidenced that 84.4% (81/85) of patients with a hug sign angle of 360° had no residual tumor at the first 1-/3-months follow-up examination. A hug sign angle of 360° also showed to be an independent protective factor against residual tumor at multivariate analysis.

**Conclusion:**

Unenhanced cone-beam CT performed at the end of a combined treatment with ablation plus chemoembolization can effectively predict an early treatment response on radiological images, when a hug sign angle of 360° was detected.

## Introduction

Hepatocellular carcinoma (HCC) represents a challenging medical condition. Personalized care of these oncologic patients is mandatory, based on a multidisciplinary evaluation of tumor characteristics, patient’s physical status, and liver function [1–3]. Among therapies for primary and metastatic liver disease, locoregional treatments represent a valuable option, and can have both curative and palliative aim [4]. Multimodal, combined locoregional approaches are known to increase treatment efficacy, preventing incomplete peripheral tumor necrosis, and improving patient survival, without increasing the complication rates [5–7]. Combined locoregional therapies that include percutaneous approaches such as radiofrequency (RFA) or microwave (MWA) ablation with trans-arterial chemoembolization (TACE) have been effectively and safely used to treat single large HCCs [8, 9]. Given the challenges in ensuring complete treatment of these larger tumors in real time, immediate prediction of residual disease after combined locoregional treatment, ideally during the procedure itself, could potentially increase the curative effect of these procedures, decreasing treatment failure rates and improving treatment of residual viable tumor, as already demonstrated for TACE and ablative techniques alone [10–15]. Recently, a new radiologic “hug sign” depicted at the intraprocedural unenhanced C-arm cone-beam computed tomography (CBCT) was described and suggested to indicate intraprocedural success [16]. However, it was noted that the predictive value of this sign needed to be confirmed in larger studies. Accordingly, the aim of our study was to retrospectively analyze the role of the hug sign obtained on intraprocedural CBCT in predicting short-term response in patients with HCC treated by combined locoregional treatments, based on the measurement of the hug sign angle.

## Materials and methods

### Study design and patient population

This study was approved by the Institutional Review Board and was performed in agreement with the 1990 Declaration of Helsinki and its amendments. All patients signed a written informed consent for the therapeutic procedure. Due to the retrospective nature of the study, patient’s informed consent to the study participation was waived. We retrospectively searched the radiological information system (RIS) database of our Institution, identifying patients with HCC treated with a single-step combined locoregional therapy consisting of thermal ablation (RFA or MWA) followed by TACE between January 2017 and September 2021. Treatment decisions were based on a multidisciplinary tumor board (MDTB) consensus obtained during dedicated meetings. Indications for combined treatment were based on the following inclusion criteria: age > 18 years, Child–Pugh score A liver cirrhosis, unresectable single large (3–7 cm) HCC or multinodular HCC with no more than three nodules and a target lesion size ranging between 3 and 7 cm, and absence of vascular invasion or extrahepatic metastases on CT or MR pre-treatment studies. All patients who underwent combined treatment had already been deemed unfit for surgical resection after MDTB evaluation due to necessity of major resection in patients with severe portal hypertension (esophageal varices graded F2 according to the Japanese Research Society for Portal Hypertension, gastric varices, splenomegaly with platelet count < 100,000/mL, or current/previous ascites), unfeasible surgery due to lesion location, severe comorbidities, or patient refusal. Exclusion criteria for combined treatment consisted of age < 18 years, pregnancy or child-bearing potential, allergy to iodinated contrast medium or local anesthesia, low platelet count (< 45,000/μL), and impaired coagulation status (International Normalized Ratio – INR > 1.5). Allergy to iodinated contrast medium, low platelet count, or impaired coagulation status were considered as definitive exclusion criteria if correction (e.g. desensitizing prophylaxis, platelet infusion, vitamin K supplements) was deemed unfeasible or unsuccessful. Pre-treatment workup consisted of physical examination, laboratory tests, liver ultrasound, radionuclide bone scan, and contrast-enhanced CT of the thorax and abdomen, or contrast-enhanced liver MRI. All patients were affected by liver cirrhosis, diagnosed by histologic and/or clinical criteria (laboratory parameters, US and/or CT/MR signs). Severe portal hypertension was diagnosed by the presence of at least one of the above-mentioned criteria that excluded patients from surgical resection. HCC diagnosis was based on the guidelines in use at the time of MDTB evaluation and of combined treatment [1]. Patients with missing preprocedural and/or intraprocedural data, without post-procedural unenhanced CBCT scan, or without a 1/3-month follow-up examination were excluded from the retrospective analysis. Patients with poor quality CBCT scans due to severe image artifacts were also excluded.

### Combined treatment

All combined locoregional treatments were performed in a fully equipped angio-suite using a single-treatment approach, by the same interventional radiologist, after antibiotic prophylaxis, with continuous patient monitoring provided by an anesthesiologist responsible for administering conscious analgo-sedation. After right common femoral or left radial access, main hepatic artery angiography was performed using a diagnostic catheter, to map liver vascularization, identify arteriovenous shunts, and to assess the feeding arteries of the target HCC. The distal tract of the segmental hepatic artery feeding the target HCC lesion was superselectively catheterized using a coaxial technique with a 2.7-Fr microcatheter (Progreat; Terumo, Japan). Thermal ablation (with RFA [RF Medical, South Korea] or MWA [Amica GEN, HS Hospital Service, Italy], based on lesion size and location, decided during the MDTB on a per-patient basis) was performed under US-guidance after local anesthesia and during patient sedation. Ablation procedures were carried out using one or more applicators, with a variable procedural time, based on the chosen technique and on lesion volume, according to the manufacturer recommendations. After the ablation, the electrode-needle was withdrawn while performing a tract-ablation, and a superselective TACE (conventional, with drug-eluting beads [DEB], or with radiopaque beads) was performed. Conventional TACE protocol was most often based on chemotherapic-in-oil (CiO) technique, in which the final emulsion contains the drug (doxorubicin, 50 mg) directly mixed in lipiodol, followed by gelfoam. DEB-TACE was usually performed using 100 micron (LifePearl, Terumo, Japan) or 100–300 micron (DC-Bead, Boston Scientific, USA) beads loaded with 50 mg of doxorubicin. TACE with radiopaque beads was performed with 70–150 micron (LCBead-LUMI, Boston Scientific, USA) beads loaded with 50 mg of doxorubicin.

The time between thermal ablation completion and TACE execution was less than 5 min. Intraprocedural contrast-enhanced CBCT was performed to identify the target tumors and their arterial feeders. The procedure was considered completed when all the drug was administered and/or slow flow was observed in the tumor-feeding artery. An unenhanced CBCT scan was acquired at the end of the procedure to check the immediate results.

### Cone-beam CT imaging

CBCT technology is now wide-spread, as it is integrated in major angiographic systems (Allura XperCT, Philips Healthcare, The Netherlands; Syngo DynaCT, Siemens Healthineers, Germany). It renders a volumetric reconstruction of 2-dimensional images, obtaining satisfactory, clinically useful soft-tissue cross-sectional depiction. Over a 5-s interval, 310 projection images (at 60 frames-per-second) were acquired by the motorized C-arm at a fixed 120 kilovoltage peak (kVp) setting. The 2D images were then reconstructed into volumetric post-procedural images for a 250 × 250 × 194 mm field-of-view (matrix size 384 × 384 × 296) with a 0.6 mm voxel size. The CBCT acquisition was performed with continuous tube current modulation, obtaining an estimated radiation dose of around 3 mSv for every single acquisition, as reported by the procedural dose-report. Patients were instructed to maintain end-expiration apnea during the CBCT scanning.

### Data collection, imaging evaluation and local response assessment

All data were extracted from the electronic medical records on the Radiology Information System and Picture Archiving and Communication System. Radiological report and medical records review was performed by an interventional radiologist with 5 years of experience. Review of images was performed by two interventional radiologists with 9 and 20 years of experience, respectively. All three readers were blinded to clinical, procedural, and tumor details during data collection and image evaluation. The following data were collected: patient characteristics (age, sex, underlying hepatopathy, clinical conditions [e.g.,, liver function according to Child–Pugh scale, ECOG performance status]), tumor characteristics (largest diameter, volume, number, distance and relationship with adjacent anatomical structures as well as vascularization [i.e., hypervascular or hypovascular lesions] based on CT and/or MRI imaging), and procedural details (ablation probe, TACE protocol). Lesion volume was calculated with the ellipsoid formula [17]. Technical success was defined both as correct needle placement within the target tumor, and as complete drug delivery during the TACE (or the obtainment of post-TACE flow-stasis for 10 heartbeats), with complete disappearance of tumor enhancement at post-treatment arteriography.

Unenhanced CBCT images obtained at the end of the treatment were evaluated to determine bead deposition/contrast distribution around the volume of the hypoattenuating ablation area. When any disagreements arose between the two investigators, the final decision was made in consensus.

The “hug sign” was considered as: (a) complete: beads/trapped contrast agent deposition completely surrounding the post-ablation necrotic volume; (b) partial: beads/trapped contrast agent deposition incompletely surrounding the post-ablation necrotic volume; (c) absent: no beads/contrast agent around the post-ablation necrotic volume. In case of partial hug sign, the “hug sign angle” (HUGs angle) was calculated by subtracting the measured circumference without beads/contrast agent) from 360°. Treatment area was evaluated on CBCT images, including ablative necrosis and hug sign thickness. Volume assessments were obtained by manual segmentation of the necrotic area and tumor margins per single slice. To minimize errors, a mean value of two measurements was employed. Only the target tumor treated with combined procedure was evaluated.

Oncologic response of the target lesion was assessed on contrast-enhanced CT or MRI scans acquired at 1-month or 3-months follow-up, using m-RECIST criteria [18].

Primary end-point was the assessment of a correlation between HUGs angle and early tumor recurrence. Secondary end-points were assessment of technical success, patient overall survival, and treatment complications.

### Statistical analysis

Continuous variables are reported as mean (± SD) and *p*-values were calculated with a two-tailed t-test for Gaussian continuous variables and with a Mann–Whitney U test for non-Gaussian continuous variables. Normal distribution was tested with Shapiro Wilk’s test. For categorical measures, frequencies and percentages are presented and *p*-values calculated with a squared-Chi or a two-tailed Fisher’s exact test as appropriate. Multivariate analysis was performed using a logistic regression model with residual tumor at 1-/3-months follow-up as dependent variable. The multivariable model included all variables considered in the univariate analysis with a *p*-value < 0.05; for variables included both as continuous and dichotomous, only the continuous variables were entered in the multivariate analysis due to collinearity issues. Odds ratios (OR) and 95% confidence intervals (95% CI) were reported. Statistical analysis was performed with STATA 15.1 (StataCorp LLC, USA).

## Results

### Study population

Two-hundred and sixteen patients with HCC underwent combined locoregional treatment in our Department between January 2017 and September 2021. Patients with unavailable pre-procedural (*n* = 34) or intra-procedural/CBCT (*n* = 26) data were excluded. Twenty-four patients were excluded due to missing follow-up. Patients with poor CBCT image quality (*n* = 4) due to severe image artifacts were also excluded. In total, 128 patients (mean age, 69.3 years ± 1.1; 87 men) with unresectable HCC who successfully underwent combined treatment, performing adequate unenhanced post-treatment CBCT imaging were included. The key clinicopathological characteristics of our patient and lesion cohort included in the final analysis are summarized in Table 1.

**Table 1:** Patient demographics and tumor characteristics, divided by tumor recurrence at early follow-up

Variables	No recurrence (*n* = 95)	Recurrence (*n* = 33)	Total (128)	*p*-value
Age	69.3 (± 1.3)	69.3 (± 2.3)	69.3 (± 1.1)	0.982
Female	33 (34.7%)	8 (24.2%)	41 (32.0%)	0.266
Cirrhosis	88 (92.6%)	29 (87.9%)	117 (91.4%)	0.401
Cirrhosis etiology				
N/A	7 (7.4%)	4 (12.1%)	11 (8.6%)	0.811
Alcohol	25 (26.3%)	7 (21.2%)	32 (25.0%)	
HCV	27 (28.4%)	11 (33.3%)	38 (29.7%)	
HBV	12 (12.6%)	5 (15.1%)	17 (13.3%)	
NASH	16 (16.8%)	5 (15.1%)	21 (16.4%)	
NAFLD	8 (8.4%)	1 (3.0%)	9 (7.0%)	
Child–Pugh				
A5	55 (57.9%)	13 (39.4%)	68 (53.1%)	0.039
A6	17 (17.9%)	13 (39.4%)	30 (23.4%)	
B7	23 (24.2%)	7 (21.2%)	30 (23.4%)	
MELD	9 (8 – 10)	9 (8–10)	9 (9–11)	0.376
Multinodular	25 (26.3%)	11 (33.3%)	36 (28.1%)	0.440
Target lesion diameter	45.2 (± 1.5)	53.2 (± 2.9)	47.3 (± 1.4)	< 0.01
Largest lesion diameter > 5 cm	19 (20.0%)	24 (72.7%)	43 (33.6%)	< 0.01
Target lesion volume	44.6 (± 4.5)	66.9 (± 9.6)	50.3 (± 4.5)	< 0.01
Hypovascular lesion	14 (14.7%)	2 (6.1%)	16 (12.5%)	0.194
Distance from capsule	6.4 (± 0.7)	5.7 (± 1.0)	6.3 (± 0.5)	0.229
Parenchymal distance from capsule	64 (67.4%)	20 (60.6%)	84 (65.6%)	0.481
Distance from vessels	5.4 (± 0.6)	3.5 (± 0.8)	4.9 (± 0.5)	< 0.01
Distance from vessels ≥ 5 mm	55 (57.9%)	11 (33.3%)	66 (51.6%)	0.015
INR	1.0 (± 0.1)	1.0 (± 0.1)	1.0 (± 0.1)	0.785
Albumin	3.8 (± 0.6)	4.3 (± 1.6)	3.9 (± 0.7)	0.552
Bilirubin	1.0 (± 0.1)	1.1 (± 0.1)	1.0 (± 0.1)	0.470
PLT	126.4 (± 11.2)	127.9 (± 16.6)	126.8 (± 9.3)	0.893
Creatinine	1.0 (± 0.1)	0.9 (± 0.1)	1.0 (± 0.1)	0.054
AST	58.0 (± 7.0)	50.0 (± 7.1)	55.9 (± 5.5)	0.207
ALT	59.0 (± 6.8)	47.6 (± 9.9)	56.0 (± 5.7)	0.087
AFP	105.9 (± 60.2)	114.9 (± 74.1)	108.2 (± 48.5)	0.873
RFA	67 (70.5%)	20 (60.6%)	87 (68.0%)	0.293
TACE				
TAE	4 (4.2%)	3 (9.1%)	7 (5.5%)	0.463
cTACE	14 (14.7%)	7 (21.2%)	21 (16.4%)	
DEB	68 (71.6%)	19 (57.6%)	87 (68.0%)	
Radiopaque	9 (9.5%)	4 (12.1%)	13 (10.2%)	

*HCV*: Hepatitis C Virus; *HBV*: Hepatitis B Virus; *NASH*: Non-Alcoholic Steato-Hepatitis; *NAFLD*: Non-Alcoholic Fatty Liver Disease; *INR*: International Normalized Ratio; *PLT*: platelets; *AST*: Aspartate Aminotransferase; *ALT*: Alanine-Aminotransferase; *AFP*: alpha-fetoprotein; *RFA*: Radiofrequency Ablation; *TACE*: Transarterial Chemoembolization; *cTACE*: conventional TACE; *DEB*: Drug-Eluting Beads

### Target lesion, procedural results, and hug sign evaluation

The mean target lesion diameter was 47.3 (± 1.5) mm, while the mean distance from vessels was 4.9 (± 0.5) mm. Treatment characteristics are summarized in Table 2.

**Table 2:** Treatment characteristics, divided by tumor recurrence at early follow-up

Variables	No recurrence (*n* = 95)	Recurrence (*n* = 33)	Total (128)	*p*-value
Minor complications > 24 h	31 (32.6%)	12 (36.4%)	43 (33.6%)	0.696
HUGs angle				
< 180°	2 (2.1%)	10 (31.3%)	12 (9.4%)	< 0.01
180°–269°	4 (4.2%)	9 (28.1%)	13 (10.2%)	
270°–359°	9 (9.4%)	9 (28.1%)	18 (14.1%)	
360°	81 (84.4%)	4 (12.5%)	85 (66.4%)	
Treatment area	55.5 (± 1.4)	59.2 (± 2.5)	56.4 (± 1.3)	0.012
Treatment volume	77.1 (± 6.0)	93.8 (± 11.4)	81.4 (± 5.5)	< 0.01

Regarding the ablation portion of the combined treatment, 87 patients underwent RFA (68.0%), with the remainder (32.0%) underwent MWA. An overall technical success of the combined treatment was achieved in 100.0% of patients. Post-procedural angiography showed a complete disappearance of the tumor vasculature/stain in all cases. A mean procedural time of 64 (± 13) minutes was recorded. No major complications occurred. Minor complications (grade 1 and 2 according to CIRSE classification) were transitory increase in serum transaminases (compared to baseline values; 39 patients, 30.4%), and transient cholecystitis (4 patients, 3.1%) [19]. None of the patients experienced a worsening of the Child–Pugh score at 1-month clinical follow-up examination.

**Fig. 1:**
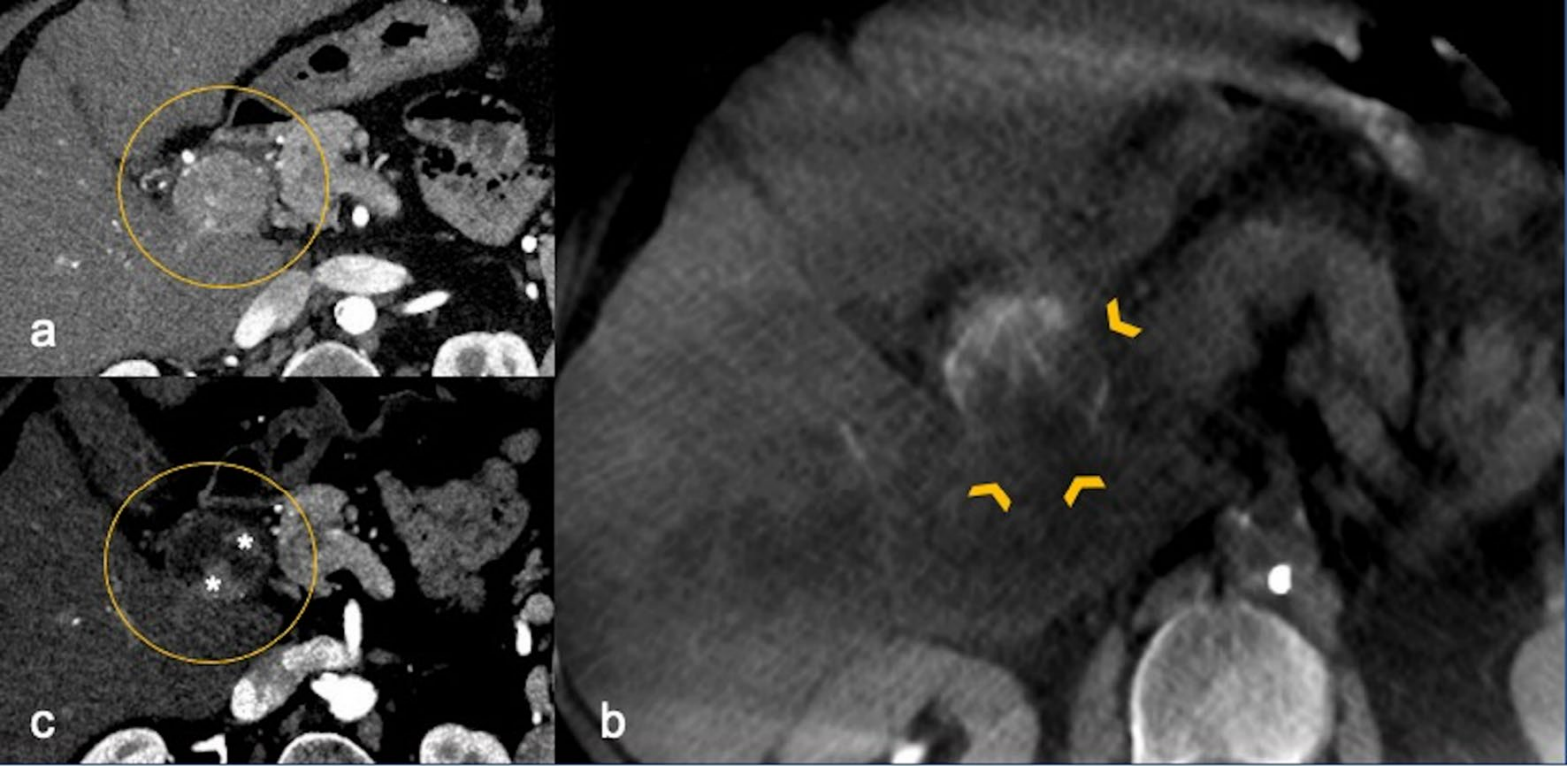
70-year-old male. **a** Pre-procedural axial arterial phase of contrast-enhanced CT scan showing a 4.3 cm exophytic HCC (circle) in IV hepatic segment. The patient underwent combined treatment: (**b**) the axial unenhanced CBCT image shows a partial Hug Sign–beads/trapped contrast agent incompletely surrounding the post-ablation volume (180°) (arrowheads). **c** 2-months follow-up axial arterial phase contrast-enhanced CT scan showing eccentric residual tumor (*) consistent with partial response. HCC: hepatocellular carcinoma. CBCT: cone-beam CT

**Fig. 2:**
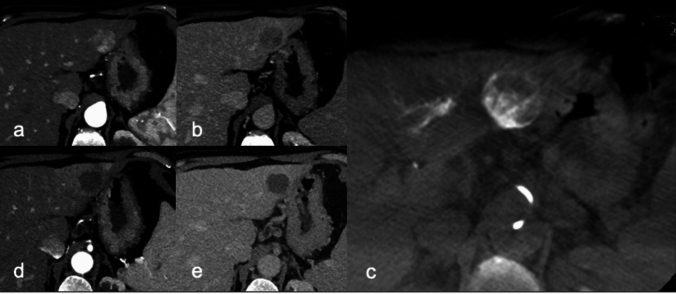
67-year-old male. Pre-procedural axial arterial (**a**) and delayed (**b**) phase of contrast-enhanced CT scan showing a 3.7 cm HCC between II-III hepatic segment. The patient underwent combined treatment: (**c**) the axial unenhanced CBCT image shows a complete Hug Sign—beads/trapped contrast agent completely surrounding the post-ablation volume. 3-months follow-up axial arterial (**d**) and delayed (**e**) phase contrast-enhanced CT images showing a complete response, without residual tumor. HCC: hepatocellular carcinoma. CBCT: cone-beam CT

The evaluation of unenhanced CBCT images obtained at the end of the treatment revealed the presence of a HUGs angle < 180° in 12 patients (9.38%), between 180°and 269° in 13 patients (10.16%) (Fig. 1), between 270°and 359° in 18 patients (14.06%) and equal to 360° in 85 patients (66.41%) (Fig. 2). The mean extent of the treatment area was 56.4 (± 1.3) cm^2^, while the mean volume was 81.39 (± 5.5) cm^2^.

### Treatment response and residual tumor

At 1–3 months follow-up examination, residual tumor consistent with partial response (PR) to the combined treatment was present in 33 (25.8%) patients, while a complete response (CR) obtained in 74.2% of patients.

**Fig. 3:**
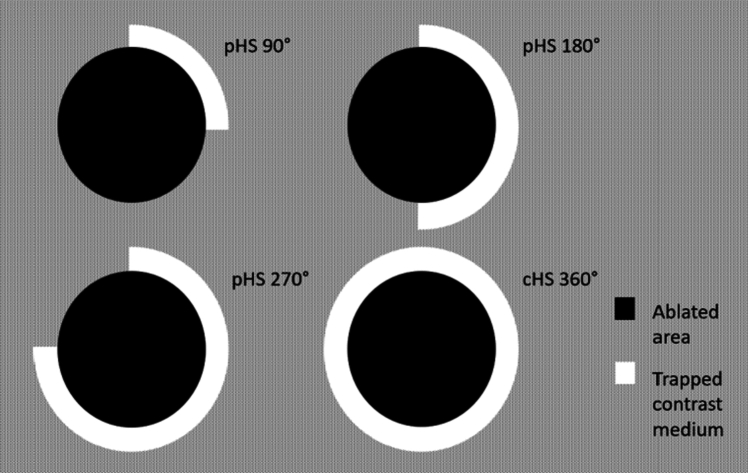
Hug Sign definition: Partial (pHS) if the deposition of the beads or of the trapped contrast agent incompletely surrounded the post-ablation necrotic volume—with angle definition obtained by deducting the degree of circumference without bead deposition or trapped contrast agent from 360°. Complete (cHS) if the deposition of the beads or the trapped contrast agent completely surrounded the post-ablation necrotic volume

**Fig. 4:**
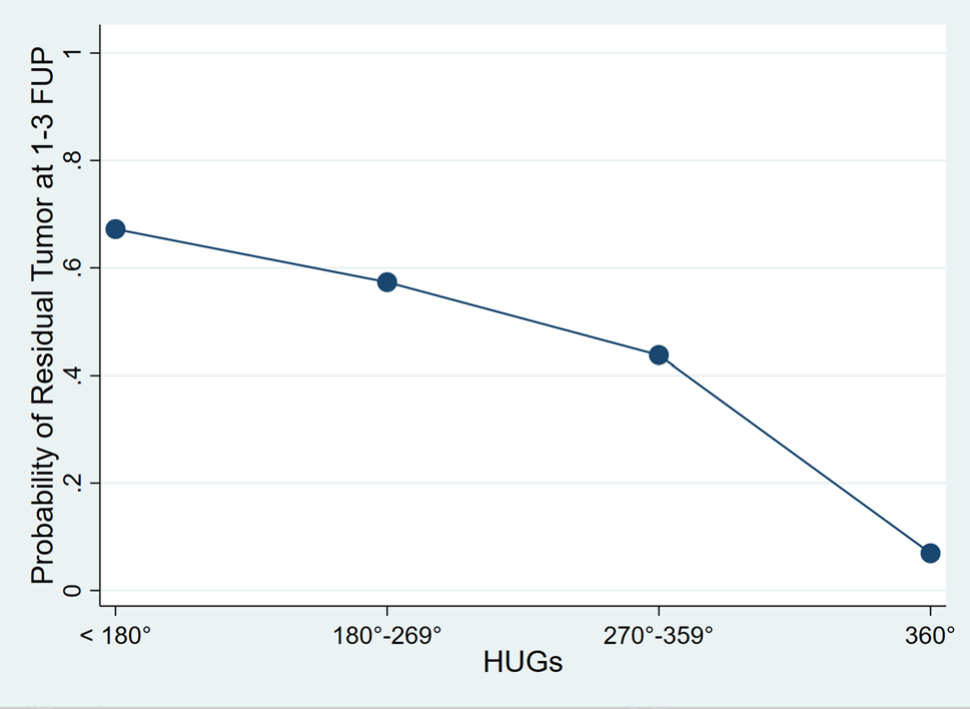
Predictive margins of residual tumor at 1-/3-months follow-up imaging, showing the reduction of residual tumor probability as the HUGs angle increases

Univariate analysis showed an association between presence of residual tumor and worse baseline Child–Pugh score (*p* = 0.039), larger target lesion diameter (*p* < 0.01), target lesion volume (*p* < 0.01), smaller distance from vessels, larger treatment area (*p* < 0.01) and treatment volume (*p* < 0.01), and lower HUGs angle (*p* < 0.01). In detail, lesions with CR had a mean diameter of 4.5 cm, whereas lesions with partial response were significantly larger, with a mean diameter of 5.3 cm and > 70.0% larger than 5 cm in size. The multivariable model (Table 3) included Child–Pugh score, target lesion diameter, distance from vessels, treatment area and HUGs angle as independent variables. Child–Pugh class A6 [OR 6.26; *p* = 0.03 (ref. class A5)] was a statistically significant predictor of residual tumor. HUGs angle of 360° [OR 0.04; *p* = 0.01 (ref. < 180°)] was an independent protective factor against residual tumor (Fig. 3). Figure 4 shows the schematic construction of the HUGs angle around the HCC lesion for statistical measurements.

**Table 3:** Logistic regression of residual tumor at 1–3 months follow-up

Variables	OR	*p*-value	[95% CI]	
Child (reference: A5)	–			
A6	6.26	0.03	1.18	33.09
B7	1.36	0.69	0.3	6.16
Target lesion diameter	1.22	0.11	0.95	1.55
Distance from vessels	01	0.98	0.73	1.36
Treatment Area	0.9	0.45	0.68	1.19
HUGs angle (reference: < 180°)	–			
180°–269°	0.58	0.63	0.07	5.21
270°–359°	0.28	0.25	0.03	2.4
360°	0.04	0.01	0.01	0.16

## Discussion

Combination locoregional treatments—using both percutaneous ablative and intra-arterial approaches synergistically—play an important role in HCC patient management, traditionally being employed to fill the gap between treating early and intermediate BCLC stages [20]. The main target of combined treatments is single large (> 3 cm) unresectable HCC. These lesions are considered BCLC-A stage, but suffer from high recurrence rates when treated with RFA even after initial CR [21]. Even when compared to MWA, which may achieve better ablative results than RFA, combined treatment obtains greater overall survival and progression-free survival rates [22]. Multiple studies have shown better results of combined treatments in terms of CR and recurrence rates when compared to ablation or chemoembolization alone [23]. Moreover, the combined treatment performed in a “single-step” fashion allows to overcome the main limitation of ablation therapies, including difficult to treat, or complex lesion location (near the diaphragm, gallbladder, and bowel) and bleeding, as performing TACE after ablation grants prompt treatment of post-ablation bleeding [5].

In concordance with previous studies, our results confirm that combined therapy with thermal ablation followed by TACE is safe, with an overall response rate of 100% and a CR rate of 75% [24, 25]. Univariate and multivariate analysis showed an association between residual tumor at first follow-up and lesion diameter and volume, as also demonstrated in previously published papers [26, 27].

It was also confirmed that location and size can also significantly influence immediate treatment response. Lower distance from vessels (less than 5 mm) was correlated with higher risk of residual tumor, mainly due to heat-sink effect despite the combined approach [28].

In contrast with previously published papers, even though MWA allows coverage of larger volumes with higher temperatures than RFA, we found no significant differences in terms of immediate local tumor response [28]. This can be explained either by even greater synergy seen between TACE and RFA or by the retrospective nature of the study, with MWA mainly used for larger lesions and in more complex location. No differences were found between various TACE techniques used, even if it is well known that distribution of contrast medium and drug is influenced by TACE modality used.

The ability to predict treatment response is of great interest. Most studies evaluated pre-procedural predictive factors, largely relying on blood tests [29, 30]. Long et al. prospectively evaluated the role of lymphocyte-to-monocyte ratio > 4 and platelet-to-lymphocyte ratio < 100 in the peripheral blood prior to the treatment as predictive factor of better local tumor control and overall survival [29]. Yamada and colleagues retrospectively investigated the role of albumin-to-bilirubin ratio prior to a sequential combined treatment [30].

Preoperative identification of patients with high risks for incomplete treatments can offer a potentially appealing real-time method to guide treatment selection, procedural aspects, postoperative monitoring, and treatment intervention. Indeed, the ability to intra-procedurally predict the treatment efficacy and the chance of CR is something that virtually every interventional radiologist would enjoy having at their disposal during a therapeutic procedure, as it could change patient’s management and prognosis.

In their combined MR and interventional radiology suite, Wang et al. showed that tumor perfusion during chemoembolization evaluated at immediate postoperative imaging was associated with greater transplant-free survival and suggested this as an imaging biomarker. However, this study is related to TACE alone procedures, and its clinical application requires dedicated hybrid MR/angio-suite and equipment [10]. Contrast-enhanced ultrasound can be useful in monitoring post-ablation treatment response [31]. Yet, contrast-enhanced ultrasound is limited by false negative results secondary to deep located HCCs or by the persistence of hyperemic halos around ablated tissue due to post-ablation inflammatory reactions. Other authors have endeavored to improve the intraprocedural prediction of ablation efficacy and the assessment of ablative safety margin using a FDG-PET-CT-guided ablation [11, 12]. Most recently, it was demonstrated that CBCT could be a useful tool for assessing the efficacy of TACE in the intra-operative setting, being a predictor of CR in conventional and DEB-TACE [13–15].

To the best of our knowledge, this is the first study to perform a systematic qualitative evaluation of unenhanced CBCT at the end of combined treatments with predictive purposes of treatment response assessment.

In cases of combined treatment consisting in ablation followed by TACE, chemotherapy drugs tend to accumulate in the peripheral portion of the lesion, as the central portion is necrotic and avascular due to the prior ablation [5]. This feature can be easily depicted in post-treatment CT images if conventional TACE with lipiodol or DEB-TACE with radiopaque beads are performed [16].

We demonstrated that unenhanced post-procedural CBCT can highlight bead placement and tumor coverage, as well as the success of embolization procedure, even without radiopaque beads. More specifically, contrast media trapped in the beads was clearly depicted by intraprocedural non-contrast CBCT images in the peripheral portion of HCC lesions around the necrotic area, like a hug. This radiological sign was called “hug sign”, showing that trapped contrast medium/lipiodol/beads completely surround the volume of ablation-related central necrosis, increasing the safety margin of the ablation procedure. With further validation, the hug sign, including hug angle evaluation, could set a standard for intra-procedural prediction of treatment efficacy and residual rates during combined locoregional treatments. Indeed, our study showed that 84.4% of patients with a HUGs angle of 360° had no residual tumor at the first follow-up CT/MR examination. This can be attributed to the well-known crucial role played by sufficient safety margin obtained for a successful combined treatment and the prevention of residual tumor tissue. In clinical practice, every interventional radiologist could benefit from an intra-procedural predictive sign of treatment success, like the hug sign, which could also warrant better and more personalized patient care and prompt retreatment (e.g.,, redo ablation or chemoembolization) in case of unsatisfactory results of the combined treatment based on the degrees of the hug sign angle. It may also represent an early predictive factor of procedural success or failure, as an interruption in the hug sign (i.e., lack or paucity of concentric contrast medium trapped/lipiodol/beads around necrotic area) is strictly related to untreated/residual viable tumor. A predictable and controlled necrosis could provide real-time feedback during embolization, being useful for decisions to stop embolization or to search for additional feeding vessels, in cases of HUGs angle lower than 360°. Our study also found that a Child–Pugh class of more than A5 was significantly correlated with a lower tumor response.

The main limitation of our paper is related to the retrospective single-center design. Our results need to be validated in larger and multicentric studies. Moreover, due to the retrospective nature of the study, the study population was inhomogeneous, both in terms of locoregional procedures performed, of tumor stage and of previous treatments. Furthermore, tumor characteristics were significantly different between recurrence and non-recurrence groups, with larger tumors in the recurrence group. This aspect could potentially lead to biased findings. Another potential limitation—beyond the aim of this study—is the lack of correlation between hug sign and overall survival. However, we intend to evaluate this in future studies. Also, we acknowledge that the hug sign is only applicable for combined treatment with ablation followed by TACE. Thus, the applicability of these criteria to combined treatment with TACE followed by ablation must be demonstrated in further studies. In addition, the short-term of our follow-up could be considered a limitation, as it does not give us the ability to understand how long in the future can the HUGs angle predict treatment response. Nevertheless, this limitation will also be hopefully overcome in future studies.

## Conclusions

This study showed that unenhanced cone-beam CT performed at the end of combined locoregional treatment with thermal ablation followed by transarterial chemoembolization can be an excellent predictor of early radiological treatment response, as a complete hug sign angle is a significant predictor of early complete response, while an incomplete hug sign angle can help the interventional radiologist in deciding whether to perform additional treatment on the spot as part of the initial treatment session.
